# A sophisticated fracture classification system of the proximal femur trochanteric region (AO/OTA-31A) based on 3D-CT images

**DOI:** 10.3389/fsurg.2022.919225

**Published:** 2022-08-31

**Authors:** Shi-Min Chang, Zhen-Hai Wang, Ke-Wei Tian, Gui-Xin Sun, Xin Wang, Yun-Feng Rui

**Affiliations:** ^1^ Department of Orthopedic Surgery, Yangpu Hospital, Tongji University, Shanghai, China; ^2^ Department of Traumatic Orthopedic Surgery, Yantaishan Hospital, Yantai, China; ^3^ No.1 Department of Hip Injury and Disease, Luoyang Orthopedic-Traumatological Hospital of Henan Province (Henan Provincial Orthopedic Hospital), Luoyang, China; ^4^ Department of Trauma Surgery, Dongfang Hospital, Tongji University, Shanghai, China; ^5^ Department of Orthopedic Surgery, Tongji Hospital, Tongji University, Shanghai, China; ^6^ Department of Orthopedic Trauma, Zhongda Hospital, Southeast University, Nanjing, China

**Keywords:** fracture classification, fracture types, trochanteric fracture, hip fractures, femur

## Abstract

**Objective:**

Fracture classification evolves dynamically with new and enhanced imaging modalities. This paper aims to introduce a novel hypothesis of a sophisticated fracture classification system for the proximal femur trochanteric region (AO/OTA-31A) based on 3D-CT images and accommodate the clinical requirement of the worldwide outbreak of geriatric hip fractures with large amounts of surgical operations.

**Methods:**

In the current practice of widely preoperative 3D-CT application and cephalomedullary nailing, we attempt to propose a new comprehensive classification system to describe the fracture characteristics in a more detailed and sophisticated architecture, and pay the most important concern to the parameters that contribute to fracture stability reconstruction in osteosynthesis.

**Results:**

The new four-by-four comprehensive classification system, followed the structure of the AO/OTA system, incorporates many fracture characteristics as dividing indexes into multiple grade levels, such as fracture line direction, the number of fragments, the lesser trochanter fragment and its distal extension (>2 cm), the posterior coronal fragment and its anterior expansion (to the entry portal of head–neck implant at the lateral cortex), the lateral wall and anterior cortex fracture, and the anteromedial inferior corner comminution. From a panoramic perspective, there are four types and each type has four subtypes. A1 is simple two-part fractures (20%), A2 is characterized by lesser trochanter fragment and posterior coronal fractures (62.5%), A3 is reverse obliquity and transverse fractures with complete lateral wall broken (15.5%), and A4 is medial wall comminution which further lacks anteromedial cortex transmission of compression force (2%). For subtypes, A2.2 is with a banana-like posterior coronal fragment, A2.4 is with distal cortex extension >2 cm of the lesser trochanter and anterior expansion of the posterior coronal fragment(s) to the entry portal of head–neck implants, A3.4 is a primary pantrochanteric fracture, and A4.4 is a concomitant ipsilateral segmental fracture of the neck and trochanter region.

**Conclusion:**

Classification represents diversity under consistency. The four-by-four sophisticated classification system delineates fracture characteristics in more detail. It is applicable in the time of rapid outbreak of trochanteric fractures in the older population, the large amounts of surgical operations, and incorporates various rare and/or more complicated subtypes which is unclassifiable before.

## Introduction

Fracture classification serves many important purposes in fracture care, including not only nomenclature and communication, but also research, education, and a guide to treatment and prognosis. However, the arbitrary grouping of the continuous injury severity of fractures into categories also has problems, and when these problems are not recognized or accounted for, they may significantly decrease the value of classification.

Fracture classification evolves dynamically from time to time. With new and enhanced imaging modalities, more and more information and collective knowledge about fractures have changed and expanded fracture classification. Computed tomography scanning and three-dimensional reconstruction (3D-CT) are such technical modalities. CT scans have become a routine part of preoperative assessment and planning for many fractures. Nowadays, 3D-CT understanding is gradually incorporated into the existing fracture classification systems (1).

The AO/OTA comprehensive fracture classification, a universal alpha-numeric coding system presented with schematic drawings and short explanation words, is widely accepted and used throughout the world. There has been continued refinement and improvement of the system. The two organizations published their first edition in 1996 (2), second edition in 2007 (3), and third edition in 2018 (4).

**Figure 1:**
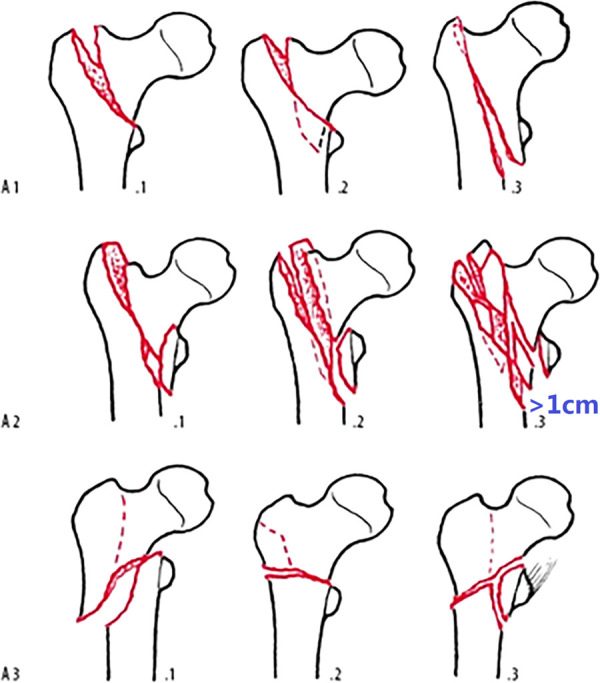
AO/OTA fracture classification of proximal femur trochanteric region 31A, 1996/2007 version.

**Figure 2:**
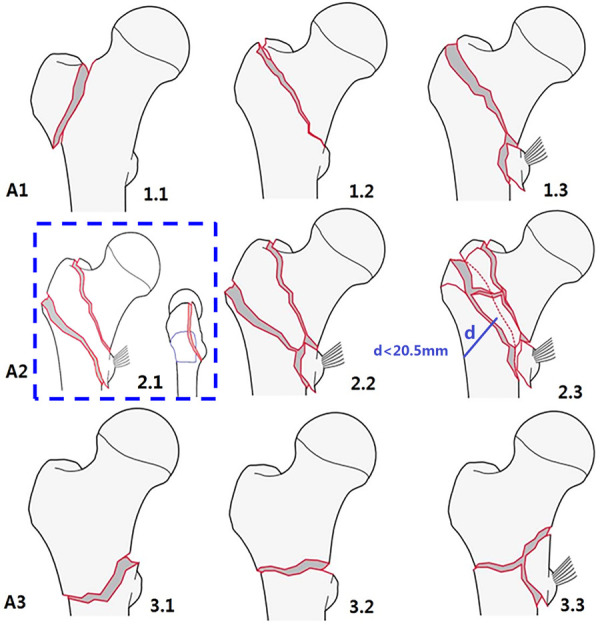
AO/OTA fracture classification of proximal femur trochanteric region 31A, 2018 version. In the blue dotted frame, the original blank of A2.1 was filled with a large banana-like posterior coronal fragment that constitutes the posterior greater trochanter, crest, and lesser trochanter with/without the posteromedial cortex (5).

Compared to the unchanged 1996/2007 version (Figure 1), the AO/OTA-2018 edition (Figure 2) completely revised the classification principles of proximal femur trochanteric region fractures (31A), which were first categorized as pertrochanteric (A1 and A2) and intertrochanteric (A3) groups according to the primary fracture line direction, separating the two main fragments, head–neck and shaft. Then the pertrochanteric fractures were further divided according to the lateral wall status into A1 simple fractures with lateral wall intact (thickness >20.5 mm), and A2 multi-fragmentary fractures with lateral wall incompetent (thickness ≤20.5 mm), while the lesser trochanter fragment was no longer used as secondary classification index in the previous 1996/2007 version. Besides the lesser trochanteric fragment, in the 2018 edition, A2.2 was characterized by one intermediate fragment (i.e., total of two free fragments and one was the lesser trochanter), and A2.3 was characterized by two or more intermediate free fragments (total ≥3 free fragments). No changes were made to the intertrochanteric A3 group, in which the lateral wall was completely broken by initial fracture. Interestingly, the position of A2.1 was blank. This emptiness makes the trochanteric region disaccord with the three-by-three classification system that was applied throughout.

Since the release of the AO/OTA-2018 edition, there were several studies on this new classification system, for example, what should be filled in the blank of A2.1 subtype (5), the accuracy and inter- and intra-observer reliability (6), and the diagnostic value of adding 3D-CT evaluation (7–11).

Based on our knowledge and experience (12–14), we want to comment on some points of the 2018 and 1996/2007 editions and propose a new comprehensive classification system to describe the fracture characteristics in a more detailed architecture.

## A critical appraisal of the AO/OTA classification

### Measurement of lateral wall thickness

The lateral wall thickness is defined as the distance in millimeters from a reference point of 3 cm below the innominate tubercle of the greater trochanter, angled at 135° upward to the fracture line on the anteroposterior radiograph (4, 15, 16). It is the mean distance between the midline of the fractured two cortical lines and the lateral cortex along the lag screw/helical blade entry route, which contains three parts, the lateral femoral cortex proper, and the remaining anterior and posterior cortices, i.e., the mean sum of the remnant length of anterior and posterior cortical wall plus the true lateral cortex thickness (17). The recommended cutoff threshold is set to be 20.5 mm. The calculated lateral wall thickness ≤20.5 mm is considered incompetent, which is directly related to the risk of fixation failure of the dynamic hip screw (DHS).

In A2 fractures, the lateral wall is partially injured by the coronal fracture line that creates the posterior coronal fragment running obliquely from the anterosuperior to the posteroinferior direction. It usually starts at the greater trochanteric apex and exits below the inferior border of the lesser trochanter, or the posteromedial cortex. This results in the remnant distance observed in the anterior cortex being always greater than that in the posterior cortex (18). Furthermore, in about 30% of cases of A2 fractures, the posterior cortex is completely destroyed and its measurement is 0, which means that the lateral femoral cortex is partially ruptured to a coronal posterior part (19).

In clinical practice, several technical problems make the measurement of lateral wall thickness not easy and not accurate based on radiographs taken in the emergency department, for example, poor quality of radiographs, difficulties in identifying the distal end of the innominate tubercle of the greater trochanter, varying degrees of external rotation of the distal femoral shaft fragment, no magnification markers on radiographs, unrecognized coronal fracture lines, different proximal femur size in sexes and ethnics, and so on.

The disagreement between measuring inaccuracy and uncertainty and the precise threshold figure (20.5 mm), makes it difficult for clinical application of the lateral wall thickness. Using CT scanning and 3D-CT reconstruction can greatly improve the measurement accuracy and enhance the agreement of classification (7–11).

### Intermediate fragments

Generally speaking, simple fractures are two-part fracture patterns (without intermediate free fragments), which creates primarily the two main fragments of pertrochanteric femur fractures, i.e., the head–neck and shaft. Stable fractures are generally simple two-part fractures. In addition, detaching a third or fourth fragment from the posterior coronal structures (the posterior part of the greater trochanter and/or the intertrochanteric crest) is still considered a stable pattern, as these upper posterior bony structures protruding beyond the posterior cortex of femoral neck, do not transmit weight-bearing force from the hip, though it is truly a multifragmented and comminuted (≥3 fragments) fracture pattern.

However, the presence of a posteromedial lesser trochanter as a third fragment is considered the most significant feature of unstable fracture patterns because it is usually larger enough and destroys the posteromedial anatomic calcar, which is the key structure of axial load transmission. Furthermore, the anterior wall or more precisely the anteromedial cortex is also an important structure for load transmission in fracture reduction.

### The discrimination ability

In the 2018 version, subtype A1.1 (isolated single trochanteric fracture) is extremely rare in the older population. Subtype A2.1 is a blank. Therefore, there are only four commonly useful subtypes for pertrochanteric A1 and A2 fractures in the 2018 classification. Compared to six subtypes in the 1996/2007 version, the discrimination ability of the 2018 edition is not as good as the 1996/2007 version.

### In the time of cephalomedullary nail

Currently, cephalomedullary nail is becoming more popular for the treatment of extracapsular hip fractures worldwide. The nail is inserted in the medullary canal, serves as a central occupation, and acts as a metallic lateral wall to support the head–neck fragment. Therefore, the value of lateral wall status (intact, incompetent, or broken) on fracture stability is decreased. On the other hand, the other two parameters, the distal cortical extension of the lesser trochanter fragment, and the anterior extension of the posterior coronal fracture line to the entry portal of head–neck implant are recognized again as predictors for postoperative un-stability after short cephalomedullary nailing (20–22).

## A novel sophisticated classification system

**Figure 3:**
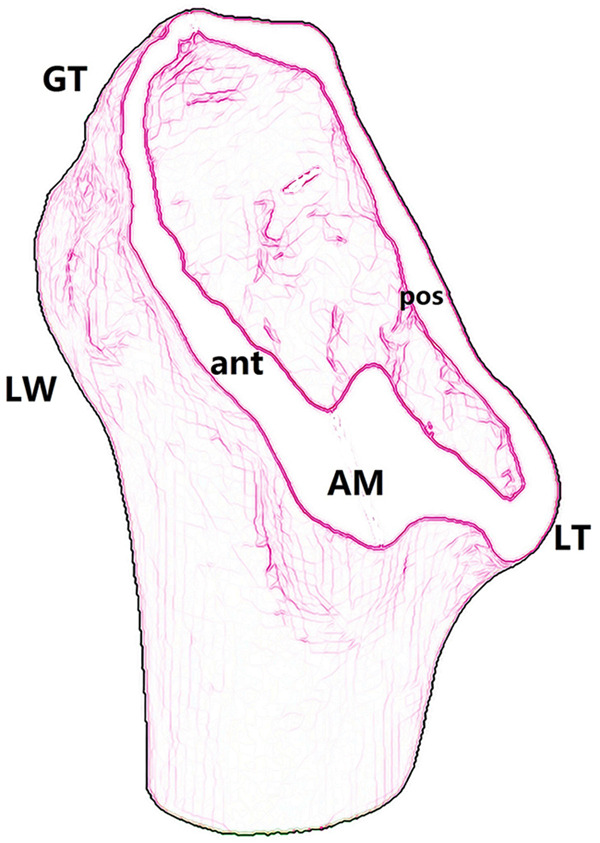
An oblique section along the intertrochanteric line to show the structures that contributes to fracture stability reconstruction: GT, greater trochanter; LT, lesser trochanter; LW, lateral wall; AM, anteromedial cortex.

**Figure 4:**
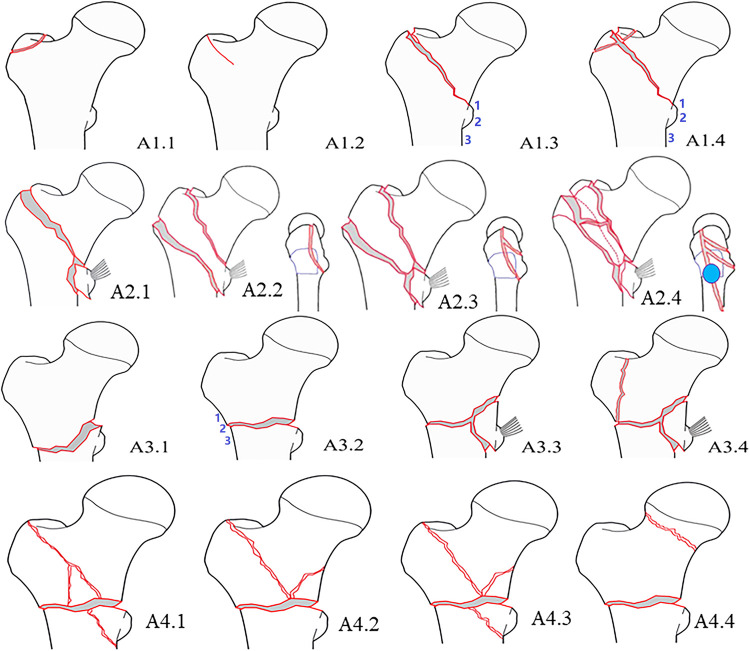
Schematic drawing to show a sophisticated fracture classification of proximal femur trochanteric region AO/OTA-31A.

Considering the architecture that contributes to fracture stability reconstruction in treatment (Figure 3), a new comprehensive and sophisticated classification system for the proximal femur trochanteric region (AO/OTA 31A) is proposed (Figure 4; Table 1). The classification system provides a panoramic perspective to the trochanteric hip fractures and combines the following factors as grouping indexes; (1) fracture line direction (standard-obliquity pertrochanteric patterns, or reverse-obliquity or transverse intertrochanteric patterns); (2) number of fragments (simple two-part, or comminuted multi-fragmentary); (3) the lesser trochanter fragment and its distal extension (>2 cm); (4) posterior coronal lateral wall and its anterior extension (involving the entry portal of head–neck implant on the lateral cortex); (5) complete lateral wall and anterior cortex transverse fracture; and (6) the anteromedial inferior corner, which is the clinical calcar cortex that transmits compressive force from the femoral head to the shaft (12–14).

**Table 1:** A sophisticated fracture classification of proximal femur trochanteric region AO/OTA-31A.

Types	Subtypes			
A1 Simple two-part fractures	A1.1 Isolated single trochanteric fracture (1) GT (2) LT	A1.2 Non-displaced fracture (1) Occult and incomplete (2) Complete with no displacement	A1.3 Two-part displaced fracture (1) LT with the shaft (2) LT with head–neck (3) LT bisected	A1.4 Two-part displaced fracture with additional posterior coronal fragment (1) Posterior part of GT (2) +Posterior crest
A2 With LT fragment, or plus partial LW fracture, posterior coronal fragment	A2.1 =1 Isolated LT	A2.2 =1 Posterior banana-like fragment (GT + LT)	A2.3 ≥2 Posterior intermediate coronal fragments	A2.4 Large posterior coronal fragments, with (1) Distal cortex extension >2 cm, (2) Anterior expansion to entry portal on lateral cortex
A3 With complete LW broken, transverse anterior cortex fracture, proximal and distal part	A3.1 Reverse oblique fracture LW fracture line is above/at/below the entry portal of the head–neck implant	A3.2 Transverse intertrochanteic fracture	A3.3 Comminuted intertrochanteric fracture	A3.4 Pantrochanteric fracture (five-part) The fracture line at the AMC inferior corner is simple and can be reduced to direct contact
A4 With further AMC inferior corner comminution, lack of medial compression force transmission structure	A4.1 Comminution of the AMC at extracapsular	A4.2 Comminution of the AMC at intracapsular, the clinical calcar	A4.3 Comminution of the AMC at both extra and intracapsular	A4.4 Segmental fracture Concomitant ipsilateral neck and trochanter fractures

GT, greater trochanter; LT, lesser trochanter; LW, lateral wall; AMC, anteromedial cortex.

As many parameters are included in this comprehensive classification, advanced image modalities are essential for accurate grouping and subgrouping, for example, MRI for pertrochanteric occult fractures. CT scanning and 3D image reconstruction provide a 360-degree full range of view, which enhances our understanding of fracture pathoanatomy and agreement of fracture classification.

Classification represents diversity under consistency. We describe the individual character diversities of subtypes under the frame consistency of types. We also depicted the novel or different patterns which is not included in the AO/OTA system.

**Figure 5:**
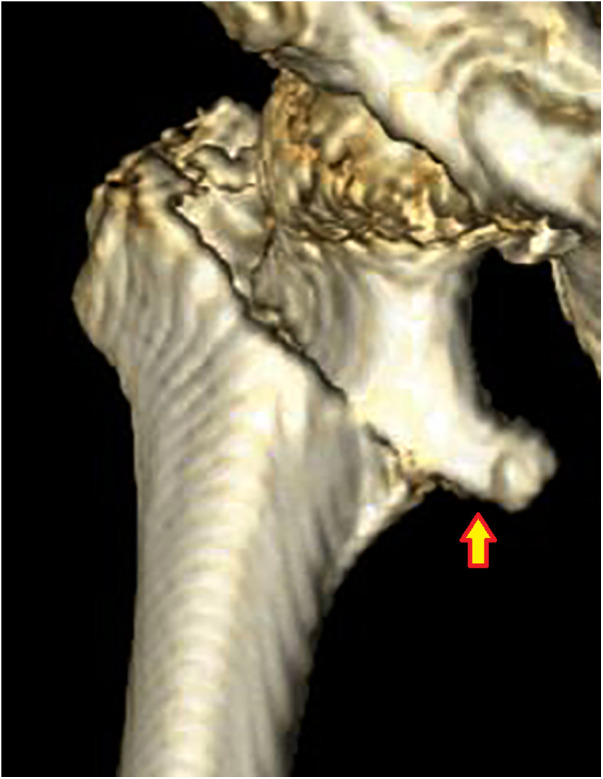
Subtype A1.3, displaced two-part fractures with lesser trochanter bisected. The arrow indicates the upper part of the lesser trochanter with the proximal head–neck fragment.

**Figure 6:**
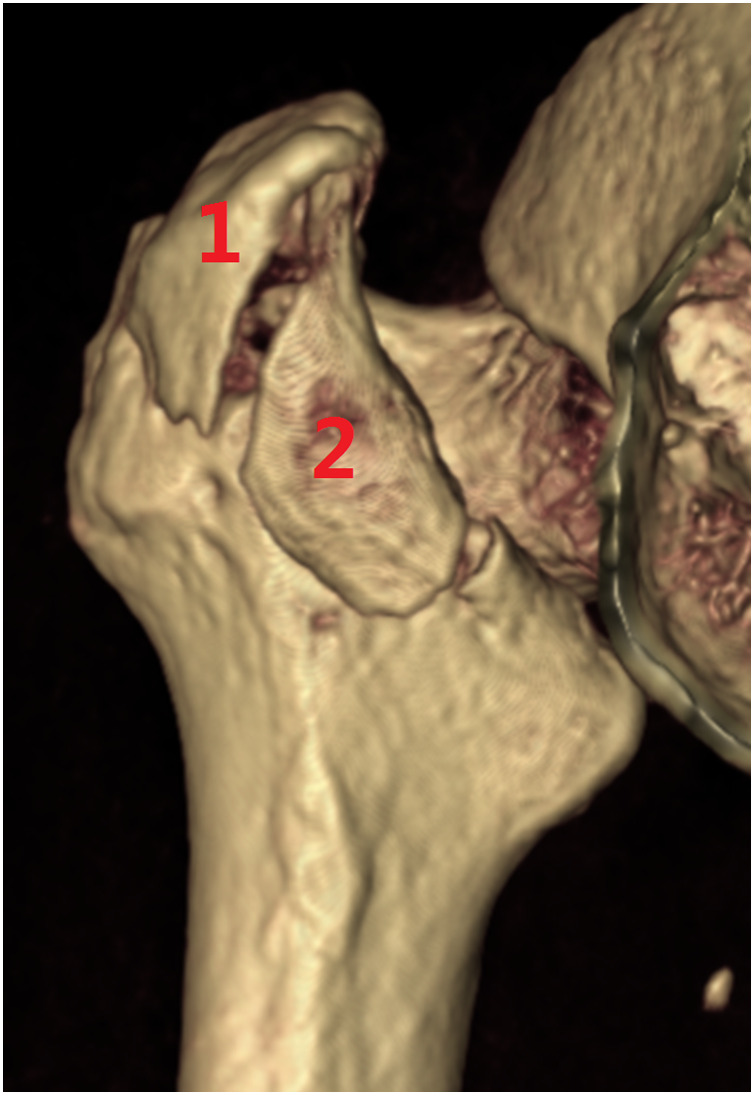
Subtype A1.4, pertrochanteric fracture with posterior greater trochanter and intertrochanteric crest fragments. These upper posterior coronal structures do not interfere with the stability between head–neck and shaft. 1 posterior greater trochanteric fragment, 2 intertrochanteric crest.

**Figure 7:**
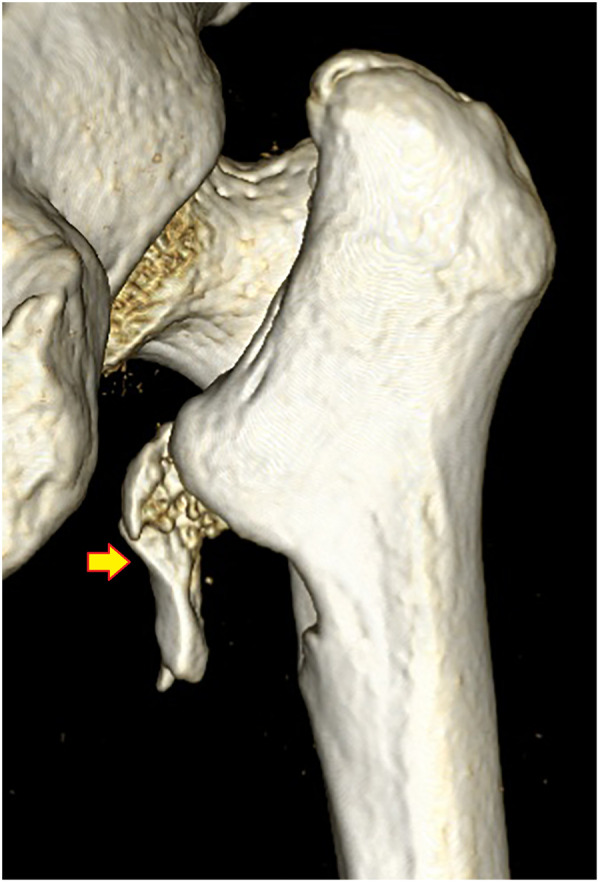
Subtype A2.1, with an isolated lesser trochanter fragment. The arrow indicates the displaced lesser trochanter.

**Figure 8:**
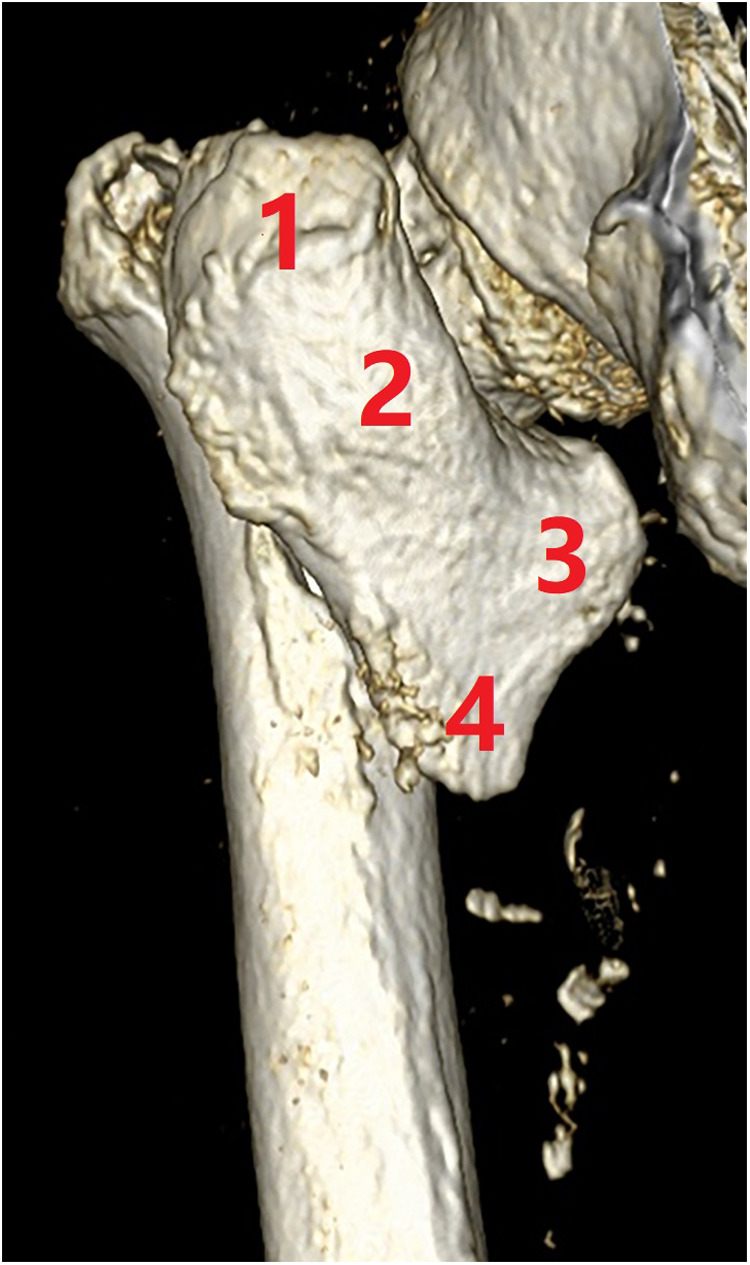
Subtype A2.2, with a large posterior banana-like fragment containing the four coronal structures. (1) Posterior greater trochanter, (2) the crest, (3) lesser trochanter, and (4) posteromedial cortex.

**Figure 9:**
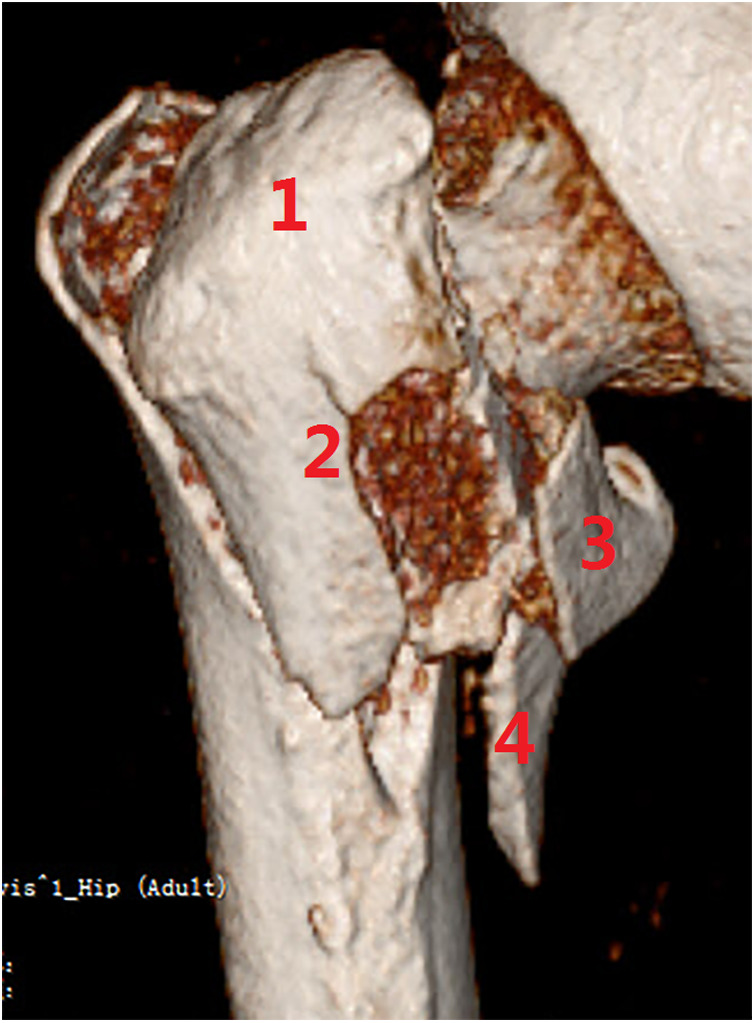
Subtype A2.3, with comminuted posterior coronal fragments (*n* ≥ 2). (1) Posterior greater trochanter, (2) the crest, (3) lesser trochanter, and (4) posteromedial cortex.

(1) Type A1 is a simple two-part pertrochanteric fracture. Subtype A1.1 is an isolated single trochanter fracture that occurred in the trochanteric region (greater trochanter or lesser trochanter). It is extremely rare in the older population and may be seen as a pathologic fracture with malignant tumor metastasis. A1.2 are incomplete fractures and nondisplaced fractures. Occult pertrochanteric fractures can be divided into three subtypes according to the length of the fracture line identified in coronal MRI and the midline axis of the femoral medullary canal, i.e., not to the midline, over the midline, and complete to the medial cortex. A1.3 is displaced two-part fractures, which can be further divided into three subgroups based on the fractured position of the lesser trochanter, i.e., lesser trochanter with proximal head–neck fragment, lesser trochanter with distal femoral shaft (basicervical fracture), and lesser trochanter bisected (Figure 5). A1.4 is further supplemented with posterior greater trochanter and/or intertrochanteric crest as a third fragment (Figure 6). However, as these upper posterior coronal structures do not interfere with the stability between head–neck and shaft and have no function in weight-bearing transmission, they are considered the same as simple two-part fractures. A1 fractures can be safely treated by dynamic hip screw/blade (DHS/DHB).

(2) Type A2 is characterized by lesser trochanter fragments and can be further complicated with different sizes and extent of posterior coronal fractures. A2.1 is a pertrochanteric fracture with an isolated lesser trochanter fragment (*n* = 1), which is not common in clinics (Figure 7). A2.2 is with a large posterior banana-like fragment (*n* = 1) that contains the three/four posterior coronal structures, i.e., the greater trochanter, the posterior crest, the lesser trochanter, and/or the posteromedial cortex (Figure 8). In a study of 154 cases, pertrochanteric fractures (types A1 and A2) (23), and posterior coronal banana-like fragments accounted for 20%, which was a common occurrence. A2.3 is with comminuted posterior coronal fragments (*n *≥ 2) (Figure 9), and there may be several combination patterns such as posterior greater trochanter and the crest as one part, the lesser trochanter, and posteromedial cortex as the other part. In our opinion, a large banana-like fragment has the same influence on stability as the comminuted posterior coronal fragments. The latter is a little bit more severe in injury degree.

**Figure 10:**
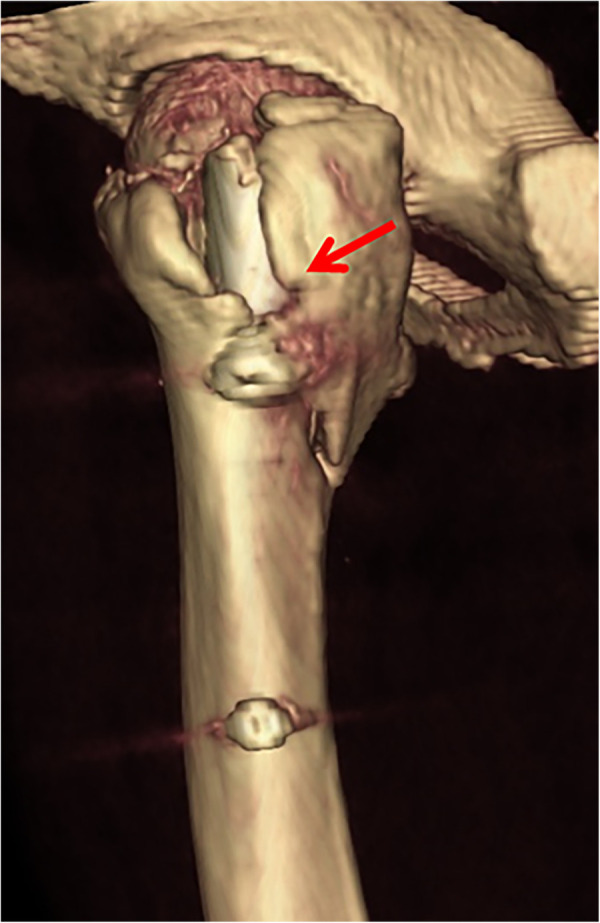
Subtype A3.4, with larger posterior coronal fragment involving the entry portal of head–neck implant at the lateral cortex. The arrow indicates an open deformity of the posterior coronal fragment by the helical blade, which may reduce nail stability in the medullary canal.

A2.4 is with more distal extension of the lesser trochanter and anterior expansion of the coronal fragment(s). The distal extension of the lesser trochanter is measured from its inferior border to the posteromedial cortex >2 cm. The anterior expansion of the posterior coronal fragment(s) is defined as the entry portal of the head–neck implant on the proximal lateral cortex. Fracture mapping studies have shown that the posterior coronal fracture line runs obliquely from the anterosuperior to the posteroinferior direction (64.6°±14.5° to the horizontal line) (18), which usually starts at the greater trochanteric apex and exits through the lesser trochanter, or the posteromedial cortex. A longer distal extension to the subtrochanteric region is usually accompanied by a wider anterior expansion on the lateral cortex. With larger posterior coronal fragment(s), the cortical circle of entry portal always becomes ruptured on the posterior part (24–27). There is an open deformity of the posterior coronal fragment(s) due to the insertion of the head–neck implant (Figure 10). In these circumstances, sagittal nail toggle or pendulum-like movement in the medullary canal may present and result in loss of fracture reduction of the anteromedial cortical apposition (14, 28).

For treatment of A2 fractures, a short cephalomedullary nail is recommended. The distal interlocking screw can be put in static or dynamic mode. However, for subtype A2.4, long nails or supplemented with cerclage wiring may be a more safe choice.

**Figure 11:**
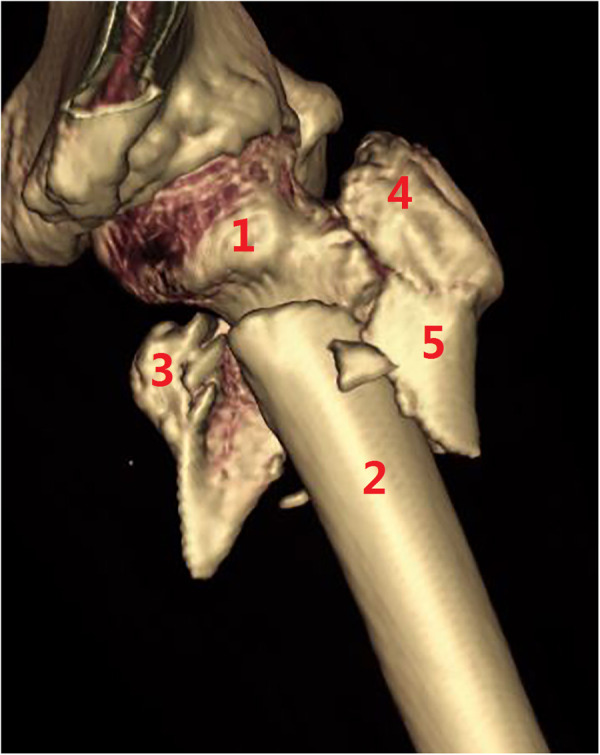
Subtype A3.4, a pantrochanteric fracture with five fragments separated. (1) Head–neck fragment, (2) femoral shaft, (3) lesser trochanter, (4) greater trochanter, and (5) lateral wall.

**Figure 12:**
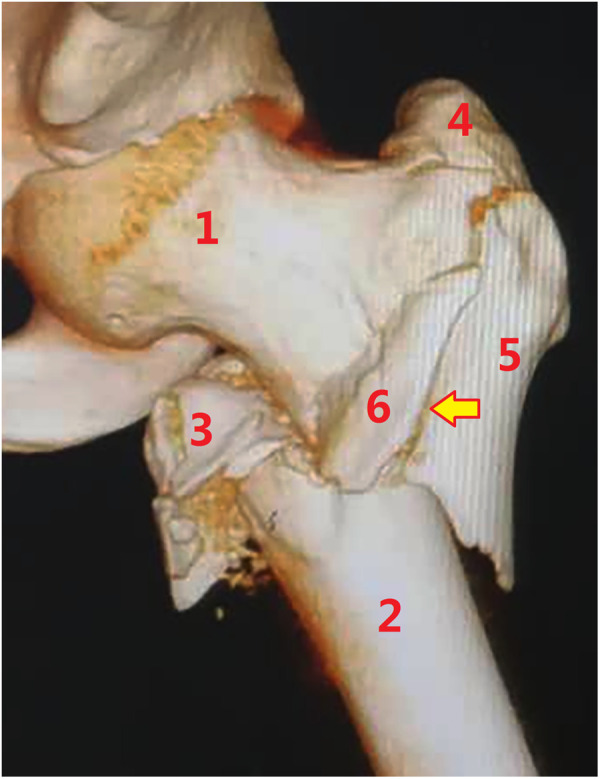
Subtype A4.1, with further comminution of the extracapsular anteromedial cortex. (1) Head–neck fragment, (2) femoral shaft, (3) lesser trochanter, (4) greater trochanter, (5) lateral cortex, and (6) anterior wall fragment.

**Figure 13:**
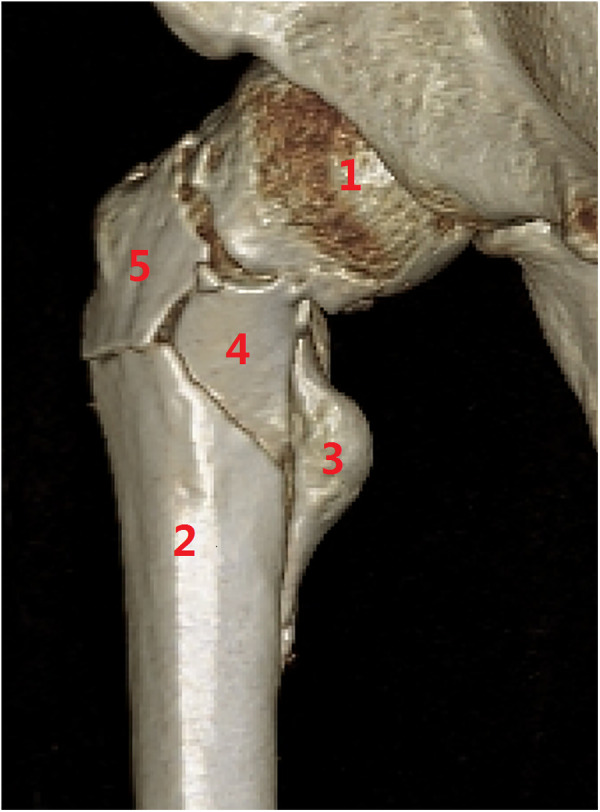
Subtype A4.2, with further comminution of the intracapsular calcar cortex. (1) Head–neck fragment; (2) femoral shaft; (3) lesser trochanter; (4) medial calcar fragment; and (5) anterior wall fragment.

**Figure 14:**
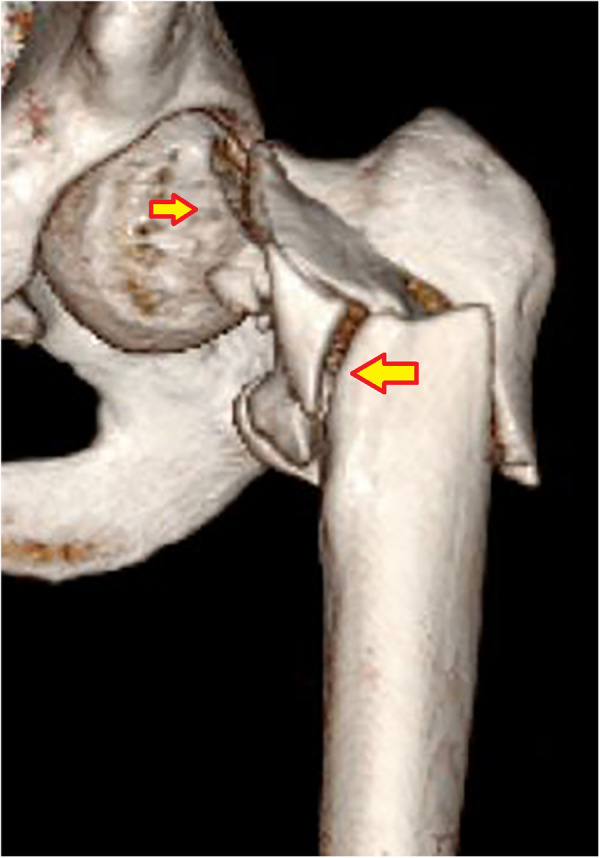
Subtype A4.4, concomitant ipsilateral segmental fractures involving both femoral neck and trochanteric region. The arrows indicate subcapital neck fracture and comminuted transtrochanteric fracture.

(3) Type A3 is reverse obliquity or transverse fractures with primary lateral wall rupture. In essence, type A3 is a complete anterior wall and lateral wall fracture, but at the anteromedial inferior corner, the fracture line is simple and leaves the opportunity to get direct cortex-to-cortex contact and support. A3.1 is with a reverse obliquity fracture line, A3.2 is with a transverse fracture line, and A3.3 is with comminuted fracture lines. A3.4 is a primary pantrochanteric fracture, resulting in separations of the five anatomic parts (Figure 11). According to the relations of the lateral wall fracture line and the entry portal of head–neck implant, three subgroups can be divided further, i.e., fracture line above the entry portal, below the entry portal (subtrochanteric), and just at the entry portal. For treatment, long cephalomedullary nails are recommended, though short or intermediate nails also can be used. The distal interlocking screw should be put in dynamic mode, and sometimes supplementary cerclage wiring should be considered.

(4) Type A4 is defined with medial comminution which lacks anteromedial load transmission architecture. It is common to lose the posteromedial lesser trochanter (and the anatomic calcar) in trochanteric hip fractures (type A2), but further comminution of the anteromedial cortex is rare (29). Lack of anteromedial cortex besides the lesser trochanter fragment means deficiency of compression force transmission structure. Subtype A4.1 is comminution with the extracapsular anteromedial cortex (Figure 12), A4.2 is comminution with intracapsular calcar cortex (Figure 13), A4.3 is comminution with both extra-capsular and intra-capsular structures, and A4.4 is a concomitant ipsilateral segmental fracture of the neck and trochanter region (30) (Figure 14). For treatment of A4 fractures, surgeons should consider combined intramedullary nails and extramedullary instrument systems, static fixation methods (plates or nails), or prosthesis replacement.

### Proportion in types and subtypes

We collected a total of 550 cases of proximal femur trochanteric fractures that received CT scanning and 3D image reconstruction. The incidence of fracture pattern is type A1 in 110 cases (20%), with A1.1 in 5 cases, A1.2 in 8 cases, A1.3 in 45 cases, and A1.4 in 52 cases; type A2 in 344 cases (62.5%), with A2.1 in 2 cases, A2.2 in 65 cases, A2.3 in 192 cases, and A2.4 in 85 cases; type A3 in 85 cases (15.5%), with A3.1 in 14 cases, A3.2 in 8 cases, A3.3 in 45 cases, and A3.4 in 18 cases, type A4 in 11 cases (2%), with A4.1 in 2 cases, A4.2 in 3 cases, A4.3 in 1 case, and A4.4 in 5 cases, respectively.

For subtype proportion, A1.3/4, A2.2/3/4, and A3.3 are relatively common. These six subtypes account for 484 cases (88%).

## Discussion

As life expectancy and elderly population increase globally, the incidence of hip fractures in the elderly, especially per/inter-trochanteric fractures, will continue to increase rapidly. Proximal femur trochanteric fractures account for about 50% of hip fractures in the geriatric population. Trochanteric femur fractures are now a global public health problem and have imposed a huge economic and social burden worldwide (31).

Fracture classification, broadly considered, is fundamental to fracture care and fracture surgery. Classifying fractures is a way to organize knowledge, transfer information, guide treatment, estimate prognosis, and enhance learning and education (1).

For the proximal trochanteric hip fractures, there have been more than ten fracture classification systems reported in the literature. Fracture classification evolves continuously with new image modalities, new knowledge compilations, and new treatment choices. We think this is especially true for the proximal femur trochanteric region (31A), as a more detailed and sophisticated system is required to fit the rapid outbreak of fracture numbers, the large amounts of surgical operations, the frequent presence of previously unclassifiable rare or more complicated patterns, and the wider applications of cephalomedullay nails.

The new four-by-four comprehensive classification system followed the logic structure of the AO/OTA system, incorporates many fracture characteristics as dividing rulers in multiple grade levels. Some new adaptations are indicated below: (1) the occult fractures are included (A1.2); (2) the posterior greater trochanter and intertrochanteric crest, the two upper structures of the coronal posterior wall, are functionless intermediate fragments (A1.4); they have no role in stability reconstruction between head–neck and shaft; (3) a large posterior coronal banana-like fragment is added to the system as subtype A2.2; (4) the distal cortical extension of the lesser trochanter >2 cm, and anterior expansion of the posterior coronal fragment(s) to the entry portal on the lateral cortex is supplemented as subtype A2.4; (5) primary pantrochanteric fracture is added as A3.4; and (6) supplement a key structure for stable fracture reduction, the anteromedial cortex (A4) (14).

The value of fracture classification lies in the accurate description of fracture characteristics, reflecting the degree of injury, guiding the choice of treatment, and helping judge the prognosis and prevent complications. Classification serves as progress of further understanding by figuring out individual characters within the framework of types and subtypes. More complicated classifications may have low reliability and reproducibility among observers. Considering the sophisticated classification system, further reliability and validity examination is needed in clinical practice. We hold promise that it will be more widely accepted and utilized, bringing orthopedic trauma surgeons closer to having a true international language on proximal femur trochanteric fractures.

## Data Availability

The original contributions presented in the study are included in the article/Supplementary Material, further inquiries can be directed to the corresponding author/s.

## References

[1] Karam MD Marsh JL. Chapter 5: classification of fractures. In: Tornetta PT Ricci WM Ostrum RF McQueen MM McKee MD Court-Brown CM, editors. Rockwood and Green's fractures in adults, 9th ed. Philadelphia: Wolters Kluver (2019). p. 104–22.

[2] Orthopaedic Trauma Association Committee for Coding and Classification. Fracture and dislocation compendium. J Orthop Trauma. (1996) 10(Suppl 1):1–154. 8814583

[3] Marsh JL Slongo TF Agel J Broderick JS Creevey W DeCoster TA Fracture and dislocation classification compendium-2007: Orthopaedic Trauma Association classification, database and outcomes committee. J Orthop Trauma. (2007) 21(10 Suppl):S1–S133. 10.1097/00005131-200711101-00001 18277234

[4] Meinberg EG Agel J Roberts CS Karam MD Kellam JF. Fracture and dislocation classification compendium-2018. J Orthop Trauma. (2018) 32(Suppl 1):S1–S170. 10.1097/BOT.0000000000001063 29256945

[5] Song H Chen SY Chang SM. What should be filled in the blank of 31A2.1 in AO/OTA-2018 classification. Injury. (2020) 51(6):1408–9. 10.1016/j.injury.2020.02.069 32093937

[6] Chan G Hughes K Barakat A Edres K da Assuncao R Page P Inter- and intra-observer reliability of the new AO/OTA classification of proximal femur fractures. Injury. (2021) 52(6):1434–7. 10.1016/j.injury.2020.10.067 33097201

[7] Isida R Bariatinsky V Kern G Dereudre G Demondion X Chantelot C. Prospective study of the reproducibility of X-rays and CT scans for assessing trochanteric fracture comminution in the elderly: a series of 110 cases. Eur J Orthop Surg Traumatol. (2015) 25(7):1165–70. 10.1007/s00590-015-1666-6 26141046

[8] Shoda E Kitada S Sasaki Y Hirase H Niikura T Lee SY Proposal of new classification of femoral trochanteric fracture by three-dimensional computed tomography and relationship to usual plain X-ray classification. J Orthop Surg. (2017) 25(1):2309499017692700. 10.1177/2309499017692700 28211303

[9] Cho YC Lee PY Lee CH Chen CH Lin YM. Three-dimensional CT improves the reproducibility of stability evaluation for intertrochanteric fractures. Orthop Surg. (2018) 10(3):212–7. 10.1111/os.12396 30152606 PMC6594481

[10] Wada K Mikami H Toki S Amari R Takai M Sairyo K. Intra- and inter-rater reliability of a three-dimensional classification system for intertrochanteric fracture using computed tomography. Injury. (2020) 51(11):2682–5. 10.1016/j.injury.2020.07.047 32718752

[11] Iguchi M Takahashi T Matsumura T Ae R Hiyama S Nakashima M Addition of 3D-CT evaluation to radiographic images and effect on diagnostic reliability of current 2018 AO/OTA classification of femoral trochanteric fractures. Injury. (2021) 52(11):3363–8. 10.1016/j.injury.2021.09.031 34598792

[12] Chang SM Zhang YQ Ma Z Li Q Dargel J Eysel P. Fracture reduction with positive medial cortical support: a key element in stability reconstruction for the unstable pertrochanteric hip fractures. Arch Orthop Trauma Surg. (2015) 135(6):811–8. 10.1007/s00402-015-2206-x 25840887 PMC4436685

[13] Chang SM Zhang YQ Du SC Ma Z Hu SJ Yao XZ Anteromedial cortical support reduction in unstable pertrochanteric fractures: a comparison of intra-operative fluoroscopy and post-operative three dimensional computerised tomography reconstruction. Int Orthop. (2018) 42(1):183–9. 10.1007/s00264-017-3623-y 28891021

[14] Chang SM Hou ZY Hu SJ Du SC. Intertrochanteric femur fracture treatment in Asia: what we know and what the world can learn. Orthop Clin North Am. (2020) 51(2):189–205. 10.1016/j.ocl.2019.11.011 32138857

[15] Hsu CE Shih CM Wang CC Huang KC. Lateral femoral wall thickness. A reliable predictor of post-operative lateral wall fracture in intertrochanteric fractures. Bone Joint J. (2013) 95-B(8):1134–8. 10.1302/0301-620X.95B8.31495 23908432

[16] Hsu CE Chiu YC Tsai SH Lin TC Lee MH Huang KC. Trochanter stabilising plate improves treatment outcomes in AO/OTA 31-A2 intertrochanteric fractures with critical thin femoral lateral walls. Injury. (2015) 46(6):1047–53. 10.1016/j.injury.2015.03.007 25890863

[17] Sun LL Li Q Chang SM. The thickness of proximal lateral femoral wall. Injury. (2016) 47(3):784–5. 10.1016/j.injury.2016.01.002 26804938

[18] Zhang Y Sun Y Liao S Chang S. Three-dimensional mapping of medial wall in unstable pertrochanteric fractures. Biomed Res Int. (2020) 2020:8428407. 10.1155/2020/8428407 32596385 PMC7285401

[19] Wei Z Xiong WF Chang SM. The remnant circumferential cortex in pertrochanteric femoral fractures: CT image study and clinical implications. Chin J Clin Anat. (2020) 38(6):639–45 (in Chinese). 10.13418/j.issn.1001-165x.2020.06.004

[20] Rehme J Woltmann A Brand A von Rüden C. Does auxiliary cerclage wiring provide intrinsic stability in cephalomedullary nailing of trochanteric and subtrochanteric fractures? Int Orthop. (2021) 45(5):1329–36. 10.1007/s00264-020-04795-4 32918572 PMC8102450

[21] Schopper C Faschingbauer M Moeller RT Gebhard F Duerselen L Seitz A. Modified candy-package technique vs cerclage technique for refixation of the lesser trochanteric fragment in pertrochanteric femoral fractures. A biomechanical comparison of 10 specimens. Injury. (2020) 51(8):1763–8. 10.1016/j.injury.2020.06.008. 32580889

[22] Lee WC Chou SM Tan CW Chng LS Yam GJM Chua THI. Intertrochanteric fracture with distal extension: when is the short proximal femoral nail antirotation too short? Injury. (2021) 52(4):926–32. 10.1016/j.injury.2020.10.059 33082031

[23] Song H Hu SJ Du SC Xiong WF Chang SM. Sub-classification of AO/OTA-2018 pertrochanteric fractures is associated with clinical outcomes after fixation of intramedullary nails. Geriatr Orthop Surg Rehab. (2021) 12:21514593211056739. 10.1177/21514593211056739. PMC8613890 34840855

[24] Li J Tang S Zhang H Li Z Deng W Zhao C Clustering of morphological fracture lines for identifying intertrochanteric fracture classification with Hausdorff distance-based K-means approach. Injury. (2019) 50(4):939–49. 10.1016/j.injury.2019.03.032 31003702

[25] Yin B He Y Wang D Zhou J. Classification of femur trochanteric fracture: evaluating the reliability of Tang classification. Injury. (2021) 52(6):1500–5. 10.1016/j.injury.2020.11.031 33280893

[26] Cho JW Kent WT Yoon YC Kim Y Kim H Jha A Fracture morphology of AO/OTA 31-A trochanteric fractures: a 3D CT study with an emphasis on coronal fragments. Injury. (2017) 48(2):277–84. 10.1016/j.injury.2016.12.015 28040260

[27] Kim YV Lee KH Lee HH Kwon GH Hwang JH Lee SW. Impact of coronal plane fragments and anterior big neck fragments on the occurrence of perioperative lateral wall fractures in AO/OTA 31-A1,2 intertrochanteric fractures treated with cephalomedullary nailing. Eur J Trauma Emerg Surg. (2022) 48. 10.1007/s00068-022-01942-x 35266020

[28] Song H Chang SM Hu SJ Du SC. Low filling ratio of the distal nail segment to the medullary canal is a risk factor for loss of anteromedial cortical support: a case control study. J Orthop Surg Res. (2022) 17(1):27. 10.1186/s13018-022-02921-z 35033125 PMC8760759

[29] Xiong WF Zhang YQ Chang SM Hu SJ Du SC. Lesser trochanteric fragments in unstable pertrochanteric hip fractures: a morphological study using three-dimensional computed tomography (3-D CT) reconstruction. Med Sci Monit. (2019) 25:2049–57. 10.12659/MSM.913593 30889172 PMC6436204

[30] Chang S Wang Z Tian K. Patterns and research progress on the concomitant ipsilateral fractures of intracapsular femoral neck and extracapsular trochanter. Zhongguo Xiu Fu Chong Jian Wai Ke Za Zhi. (2021) 35(9):1079–85 (in Chinese). 10.7507/1002-1892.202102090 34523270 PMC8444139

[31] Zhang Z Qiu Y Zhang Y Zhu Y Sun F Liu J Global trends in intertrochanteric hip fracture research from 2001 to 2020: a bibliometric and visualized study. Front Surg. (2021) 8:756614. 10.3389/fsurg.2021.756614 34778363 PMC8581155

